# Healthcare accessibility, utilization, and quality of life among internally displaced people during the Sudan war: a cross-sectional study

**DOI:** 10.1186/s13031-025-00655-3

**Published:** 2025-03-01

**Authors:** Hind Elmukashfi ShamsEldin Elobied, Muhannad Bushra Masaad Ahmed, Ahmed Balla M. Ahmed, Romaysa Abdelrahman Hassan Salih, Sohaib Mohammed Mokhtar Ahmed, Abdulhadi M. A. Mahgoub, Abdelmoula Hashim Abdelmagid Mohamed, Eman Hamid Abdallah Elamin, Al-Romaysa M. Osman Khalafalla El-Haj, Mohamed Al-Hadi Hamed Abd-Allah, Arwa Yagoub Elmadani Ibrahim, Yousuf Alnoor Younis Mohamed, Arig Elias Shabo

**Affiliations:** 1 Faculty of Medicine, Al-zaiem Al-azhari University, Khartoum, Sudan; 2 Medical Research Group, Khartoum, Sudan; 3 Faculty of Medicine and Health Sciences, University of Gadarif, Gadarif, Sudan; 4 Faculty of Medicine, University of Khartoum, Al-Qasr Street, PO Box: 102, Khartoum, 11111 Sudan; 5 Faculty of Medicine, University of Medical Sciences and Technology, Khartoum, Sudan; 6 Faculty of Medicine, University of Gezira, Wad Medani, Sudan; 7 Faculty of Dentistry, University of Gezira, Wad Medani, Sudan; 8 Faculty of Medicine, Omdurman Islamic University, Omdurman, Sudan; 9 Faculty of Medicine, Imperial University College, Khartoum, Sudan

**Keywords:** Accessibility, Utilization, Quality of life, Healthcare, Internally displaced people, Sudan, War

## Abstract

**Background:**

The ongoing war in Sudan has triggered a massive displacement crisis, leaving internally displaced people (IDP) struggling to access healthcare services. This study aimed to investigate healthcare access, utilization, and the quality of life among Sudanese IDP during the conflict.

**Methods:**

This cross-sectional study was carried out among Sudanese internally displaced people in six states. Quality of life was assessed using the WHOQOL-Bref questionnaire. Accessibility, utilization, and the consequences of not accessing healthcare were evaluated using an author-designed questionnaire, which was piloted prior to the study. Chi-square tests were used to analyze associations between categorical variables, while ANOVA was applied to assess differences in quality-of-life domains based on displacement duration and living conditions. Multinomial logistic regression identified predictors of healthcare affordability, with significance set at *p* < 0.05.

**Results:**

Among 612 participants, 40.3% reported facilities being very close, 13.0% faced waits over 2 h, and 54.3% found healthcare unaffordable. Only 33.6% always had access to qualified staff, and 22.8% of IDP visited public healthcare facilities supported by non-governmental organizations. The psychological domain had the highest quality-of-life mean score at 49.7 (18.1). Worsened symptoms (44%) were a common consequence of healthcare inaccessibility, while lack of transport (37.9%) was the most common barrier. Availability of qualified staff significantly increased the likelihood of seeking care (χ² = 11.30, *p* = 0.022). Quality-of-life domains varied significantly by displacement duration and living situation (*p* < 0.011).

**Conclusion:**

This study revealed significant variations in healthcare access and utilization among Sudanese IDP, with quality-of-life domains lower than those of IDP in other countries. Interventions should prioritize innovative solutions like telemedicine, targeted support for vulnerable groups, and expanding health insurance coverage to enhance access and long-term health outcomes.

**Supplementary Information:**

The online version contains supplementary material available at 10.1186/s13031-025-00655-3.

## Background

On April 15, 2023, intense fighting erupted between the Sudanese Armed Forces (SAF) and the Rapid Support Forces (RSF), beginning in the capital and rapidly spreading across Sudan [1]. This conflict has exacerbated the country’s pre-existing crises, including ongoing violence, disease outbreaks, and economic challenges [2]. It is estimated that around 12,501 people have lost their lives in the clashes, 24.8 million people are in need of humanitarian assistance, and approximately 9.1 million people have been displaced, marking the largest internally displaced population ever reported [3, 4].

The healthcare system in Sudan is divided between public and private sectors. The public sector operates under a framework involving multiple governmental ministries, hospitals, and clinics, while the private sector includes private hospitals, specialized clinics, and outpatient services, often complementing public healthcare provisions. International aid also plays a pivotal role, with non-governmental organizations (NGOs) and international bodies providing essential financial resources, particularly in emergencies such as epidemics or natural disasters [5]. Before the current war, Sudan’s health system was already fragmented between governmental, nongovernmental, private sector, and international actors. There was a stark geographic inequality in access to functional health facilities, qualified health workers, and lifesaving medicines. Dozens of United Nations (UN) agencies and international and national NGOs had stepped in to fill the gap with health-related humanitarian assistance [6].

The war has further strained the healthcare system, leading to severe disruptions in healthcare provision. By May 2023, 68% of hospitals in conflict-affected areas were reported as non-functional. Of the 88 hospitals in the capital and conflict-stricken states, 60 have been closed or suspended operations, leaving only 28 hospitals fully operational or partially functional. Those that have remained open are often unable to provide basic medical services due to a lack of medical staff, supplies, water, and electricity [7].

Internally displaced people (IDP), in particular, are facing numerous challenges during the war. These include limited access to water, family conflicts, heightened risks of sexual abuse and human rights violations, as well as physical and mental health issues, all of which exacerbate their social and economic vulnerabilities and increase their need for healthcare [8, 9]. Access to healthcare services is often severely limited in displacement areas due to the scarcity of healthcare facilities, professionals, and medical supplies, as well as geographic barriers and discrimination faced by IDP in host communities. These factors lead to delayed treatments and, in some cases, death [10].

The situation for IDP during the Sudan war is worsened by the devastating impact on the healthcare system, with many hospitals forced to cease operations, leaving IDP with limited access to basic medical services and significantly reducing their quality of life [11]. Access to healthcare is crucial for enhancing quality of life (QOL) by enabling early detection and management of health issues, ensuring timely and appropriate care, and providing essential support services [12]. During this war, the majority of IDP households in Khartoum, Gezira, and Northern Sudan were reported to stay with host families (87%). In contrast, IDP households in South Darfur were reported to live in camps (59%), with host families (24%), and in open area gathering sites (8%) [13]. IDP outside of camps frequently depend on informal support networks, such as host communities or extended family, for survival. These networks often lack sufficient resources to provide adequate support, which increases vulnerabilities. The UNHCR report on IDP outside of camps highlights that many IDP are “hidden” in urban settings, making it difficult for humanitarian organizations to identify and help them [14]. This dependence on informal networks can exacerbate issues like malnutrition and poor health outcomes, as seen in Sudan, where displaced families struggle to access basic necessities [15].

Despite the urgent need for information on healthcare access and its implications for health and quality of life during the Sudan war, research on this topic remains limited. To address this gap, this study aimed to explore the barriers and facilitators affecting access to healthcare services among Sudanese IDP during the current war, focusing on accessibility, utilization, and the quality of life of these vulnerable populations.

## Methods

### Study design and setting

A cross-sectional study was conducted among Sudanese internally displaced people (IDP) during the ongoing conflict in Sudan to evaluate the barriers and facilitators to accessing healthcare services. The research was carried out in six states: Gezira, Gadarif, White Nile, Northern State, River Nile, and Kassala. In Gezira State, data collection was limited to Al Managil locality, as it remained unaffected by the conflict, unlike other areas of the state. These states were selected as they represent the safest regions in eastern, northern, and central Sudan. The study’s methodology and findings are comprehensively detailed in the manuscript, following the STROBE (Strengthening the Reporting of Observational Studies in Epidemiology) guidelines [16].

### Study population

Data were collected from internally displaced Sudanese individuals living in IDP camps or nearby communities. Participants in the camps were recruited through camp supervisors, while those in the communities were approached at hospitals and centers that serve IDP. Additionally, schools with IDP populations were visited for recruitment. The study excluded individuals under the age of 18 and those who declined to participate.

### Sampling

The minimum sample size for the study was calculated using the Cochran formula [17], yielding 384 participants. This calculation was based on a 95% confidence interval, a 5% margin of error, and an expected prevalence of 50%, reflecting an unknown target population size. Due to the use of a non-probability sampling method and the absence of updated official records on the total IDP population in the target states, adjustments were made to ensure adequate representation. A 10% increase was added to account for potential non-responses, raising the sample size to 422 participants. Additionally, an over-sampling factor of 1.5 was applied to account for population heterogeneity and enhance generalizability, resulting in a final adjusted sample size of 633. Convenience sampling was used due to the lack of access to official IDP records.

### Data collection tool and procedure

Data were collected using a face-to-face interview questionnaire comprising five sections. The first section captured demographic characteristics of IDP. The second section assessed quality of life across four domains—physical, psychological, social, and environmental—using the WHOQOL-Bref, a standardized tool adapted by the World Health Organization (WHO) [18]. The Arabic version of this questionnaire had been validated.

The third and fourth sections focused on healthcare accessibility and utilization, respectively. The fifth section examined the implications of healthcare inaccessibility, including questions about the effects of limited access on health and alternative solutions when services were unavailable. The last three sections were adapted from a study in Uganda [19]. Additional questions were added to these sections to ensure consistency.

The questionnaire was piloted with 33 IDP, and the Cronbach’s alpha coefficient for the scale was 0.545, yielding a moderate level of internal consistency. Adjustments were made based on recommendations from the pilot analysis. Participants in the pilot were excluded from the final study. Except for the second section, all parts of the questionnaire were translated into Arabic by the authors and reviewed by a translation expert.

Data collection was conducted by trained medical students from the research team. Participants were briefed about the study’s objectives, assured of confidentiality, and provided written informed consent before participation. Surveys were administered in Arabic and local dialects to accommodate linguistic preferences.

### Data analysis management

The analysis for this study was conducted using Jamovi version 2.6.19. All statistical procedures, data visualization, and summary statistics were performed using this software. Demographic and health-related variables were analyzed using descriptive statistics, including frequencies, percentages, means, and standard deviations, to summarize participant characteristics and key outcomes. Chi-square tests were employed to examine associations between categorical variables, as chi-square is appropriate for categorical data and helps assess the relationships between variables like gender, employment status, and healthcare utilization. ANOVA was utilized to evaluate differences in quality-of-life domains based on displacement duration and living situation, as it is suitable for comparing means across multiple groups of continuous data, such as quality-of-life scores. Multinomial logistic regression was applied to identify predictors of healthcare affordability. It included gender, age, highest educational level attained, current employment status, household size, number of working family members, presence of pregnant women or children under five in the household, duration of internal displacement, current living situation, duration of stay in camp/community, healthcare cost-related delays/avoidance, health insurance coverage, and healthcare utilization in the past year as independent variables. The significance levels were set at *p* < 0.05.

## Results

The response rate for this study was 97%. The study sample (*N* = 612) consisted of 66.1% females (*n* = 403) and 33.9% males (*n* = 207). The mean age was 35.1 years (SD = 14.9), with a range from 18 to 83 years. The average number of working family members was 2.0 (SD = 1.4), ranging from 0 to 12. (Table 1)

In terms of displacement, 34.7% had been displaced for more than 12 months, while 17.0% had been displaced for less than 3 months. The majority of participants lived in public buildings (36.1%), followed by host communities (22.1%) and displacement camps (19.2%). The duration of stay in these locations varied, with 39.8% staying for more than 6 months and 23.3% for less than 3 months. (Table 1). Further information on participants’ demographics as educational background and employment status could be found in Supplementary File 1.

**Table 1:** Demographic characteristics of participants

	Overall (*N* = 612)		Overall (*N* = 612)	
**Gender**		**Duration of Internal Displacement**		
Female	403 (66.1%)	Less than 3 months		102 (17.0%)
Male	207 (33.9%)	More than 12 months		208 (34.7%)
**Age**		3 to less than 6 months		150 (25.0%)
Mean (SD)	35.1 (14.9)	6 to less than 9 months		68 (11.3%)
Range	18.0–83.0	9 to less than 12 months		72 (12.0%)
**State**		**Current Living Situation**		
Gezira	118 (19.3%)	Other		138 (22.6%)
Gadarif	175 (28.6%)	Public Buildings (e.g., schools)		220 (36.1%)
White Nile	62 (10.1%)	Host Community		135 (22.1%)
Northern	166 (27.2%)	Displacement Camp		117 (19.2%)
Kassala	18 (2.9%)	**Duration of Stay in Camp/Community**		
Nile River	72 (11.8%)	Less than 3 months		140 (23.3%)
**Number of Working Family Members**		More than 6 months		239 (39.8%)
Mean (SD)	2.0 (1.4)	3 to 6 months		221 (36.8%)
Range	0.0–12.0			

The study revealed important insights into healthcare access and utilization among Sudanese IDP. Regarding the distance to healthcare facilities, 40.3% of participants reported that the facilities were “Very Close”, while 48.2% indicated they were “Neither Close nor Far”, and 11.5% found them “Very Far”. Waiting times varied, with 26.4% of respondents experiencing a wait of 1–2 h, and the majority, 34.1% reporting waits of less than an hour. In terms of transportation, the majority (53.7%) used walking to reach healthcare facilities, followed by public transport (32.1%) (Table 2).

Concerning affordability of healthcare services, 54.3% of participants indicated that healthcare was not affordable, while 37.9% found it affordable. Consequently, a significant proportion (62.5%) reported delaying or avoiding healthcare due to cost, highlighting a major barrier to healthcare access. In terms of healthcare insurance, 51.3% of respondents did not have health insurance, while 18.2% have it and it covers their health needs, 3.3% they do not know, and 27.2% have it but it does not cover their health needs. Booking appointments with healthcare providers was generally challenging, with 27.7% reporting that they sometimes couldn’t get an appointment (Table 2).

Regarding healthcare personnels, 33.6% of participants reported always having access to qualified staff, while 45.2% had staff available sometimes, and 1.8% reported never having access to qualified personnel.

The types of healthcare facilities most frequently visited included private healthcare facilities (22.3%), public facilities supported by NGOs (22.8%), and community-supported facilities (12.0%). These findings underline the varied challenges faced by IDP in accessing affordable and timely healthcare services, as well as the importance of qualified healthcare staff and accessible facilities (Table 2).

**Table 2:** Healthcare access and utilization among Sudanese IDP

	Overall (*N* = 612)
**How far is the healthcare facility from the camp/host community**?	
Very Far	70 (11.5%)
Very Close	245 (40.3%)
Neither Close nor Far	293 (48.2%)
**What means of transportation do you use to reach the healthcare facility**?	
Other	12 (2.0%)
Walking	328 (53.7%)
Public Transports	196 (32.1%)
I have not visited a healthcare facility	18 (2.9%)
Private vehicle	57 (9.3%)
**Are healthcare services affordable for you?**	
No	325 (54.3%)
I don’t know	47 (7.8%)
Yes	227 (37.9%)
**Have you delayed or avoided healthcare services due to costs?**	
No	224 (37.5%)
Yes	373 (62.5%)
**If you have health insurance**,** does it cover your services**?	
No	163 (27.2%)
I Don’t Know	20 (3.3%)
I don’t have health insurance	308 (51.3%)
Yes	109 (18.2%)
**How easy is it to book an appointment with a healthcare provider**?	
Sometimes, I can’t get an appointment	169 (27.7%)
Not easy at all	86 (14.1%)
I haven’t requested healthcare services	56 (9.2%)
Yes, it’s easy	300 (49.1%)
**Availability of qualified staff at healthcare facilities**	
Never	11 (1.8%)
Sometimes	276 (45.2%)
Always	205 (33.6%)
I Don’t Know	64 (10.5%)
Rarely	55 (9.0%)
**Waiting time to see a healthcare provider**	
Long (1–2 h)	161 (26.4%)
Not long (< 1 h)	208 (34.1%)
Very long (> 2 h)	79 (13.0%)
I haven’t visited a healthcare facility	60 (9.8%)
No waiting time	102 (16.7%)
**Where do you usually obtain prescribed medications?**	
Other	7 (1.1%)
Local pharmacy	250 (41.0%)
Healthcare facility	230 (37.7%)
Outside Sudan	7 (1.1%)
Outside the State	13 (2.1%)
I don’t take any medications	33 (5.4%)
From a nearby area	70 (11.5%)
**Informed about healthcare facilities surrounding the camp/host**	
No	195 (32.0%)
I Don’t Know	102 (16.7%)
Yes	312 (51.2%)
**Have you sought medical services in the past year?**	
No	161 (26.8%)
Yes	440 (73.2%)
**If yes**,** how many times?**	
Mean (SD)	3.2 (4.5)
**Type of healthcare facility visited**:	
Other	17 (2.8%)
Mobile Clinic	42 (6.9%)
Didn’t visit a healthcare center	65 (10.7%)
Community-supported facility	73 (12.0%)
Private healthcare facility	136 (22.3%)
Public facility supported by NGOs	139 (22.8%)
Public facility unsupported by NGOs	129 (21.1%)
Traditional/alternative medicine	9 (1.5%)

The quality of life across various domains was evaluated. In the physical domain, participants reported a mean score of 44.8 (SD = 17.3), while in the psychological domain they exhibited a slightly higher mean score of 49.7 (SD = 18.1). In the social domain, the mean score was 45.0 (SD = 24.5) (Table 3).

**Table 3:** Quality of life domains

Quality Of Life Domains			
**Physical Domain**		**Social Domain**	
Mean (SD)	44.8 (17.3)	Mean (SD)	45.0 (24.5)
**Psychological Domain**		**Environmental Domain**	
Mean (SD)	49.7 (18.1)	Mean (SD)	37.3 (17.0)

**Fig. 1:**
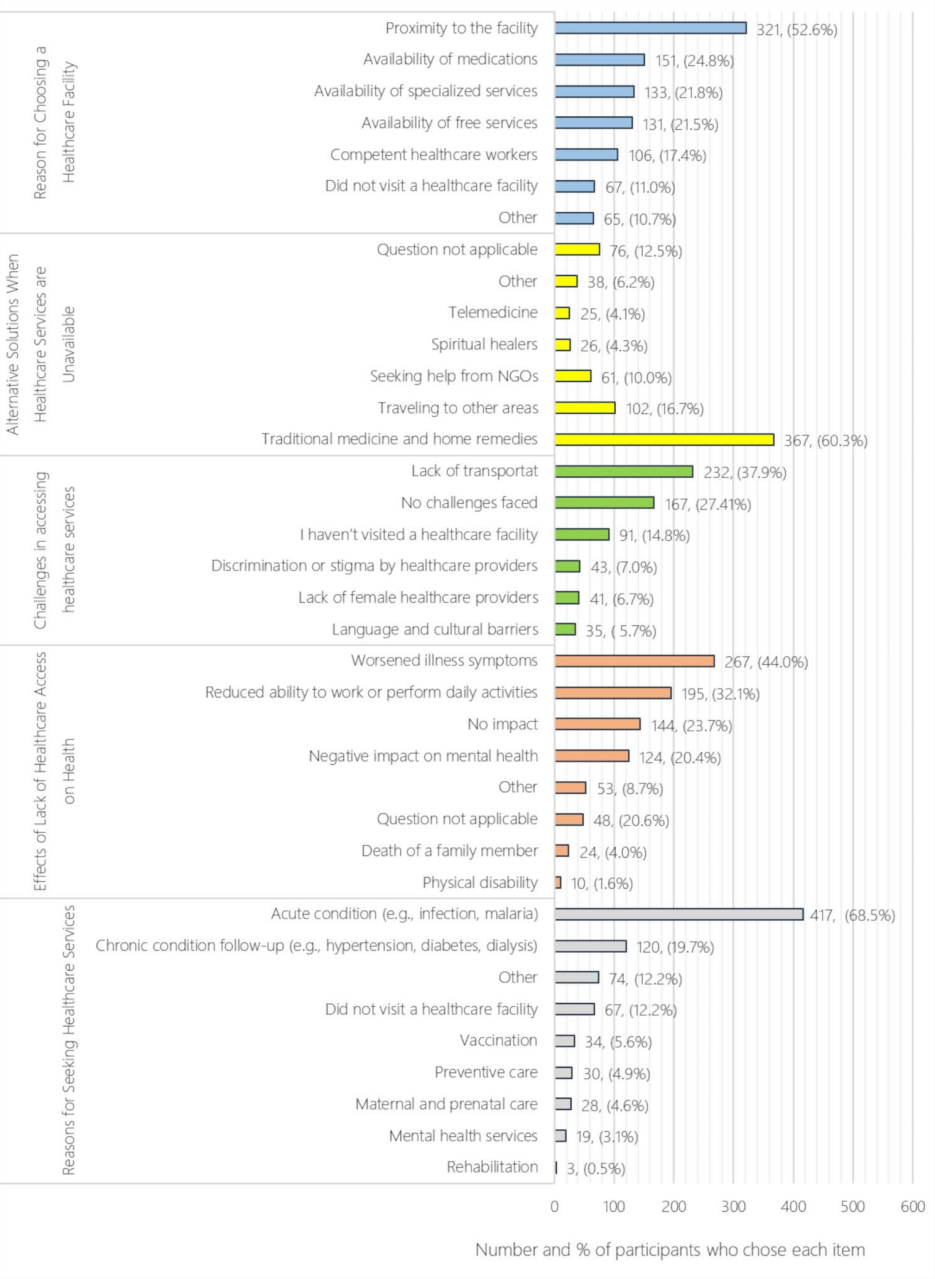
Reasons for healthcare choices, challenges in access, and consequences of unmet healthcare needs

Figure 1 reveals the primary factors influencing participants’ healthcare choices, challenges, and the impact of unmet healthcare needs. Proximity to the facility was the most common reason for choosing a healthcare center (52.6%). For unavailable healthcare services, 60.3% resorted to traditional medicine and home remedies as an alternative. Lack of transport emerged as a significant barrier to access (37.9%), though 27.4% reported no challenges. Interestingly, discrimination or stigma was also identified as a factor hindering health-seeking behaviors, accounting for 7%. Acute conditions (68.5%) were the predominant reason for seeking healthcare, with chronic conditions and preventive care being less frequent (19.7% and 4.9%, respectively). These findings underscore the critical role of accessibility and resource availability in healthcare-seeking behavior.

Multinomial logistic regression revealed significant predictors of perceived affordability of healthcare services among Sudanese IDP. These predictors included gender, where males had lower odds (OR = 0.49, 95% CI: 0.29–0.83, *p* = 0.007) compared to females. Duration of internal displacement was also significant, with those displaced for 3 to less than 6 months (OR = 0.17, 95% CI: 0.03–0.83, *p* = 0.033) and 9 to less than 12 months (OR = 0.085, 95% CI: 0.01–0.49, *p* = 0.008) having lower odds of reporting affordability. Individuals residing in displacement camps had significantly lower odds (OR = 0.10, 95% CI: 0.01–0.68, *p* = 0.013) of perceiving healthcare as affordable. Healthcare cost-related delays or avoidance were associated with lower odds (OR = 0.26, 95% CI: 0.14–0.47, *p* < 0.001), while health insurance coverage influenced affordability perceptions, with those unaware of their coverage (OR = 22.28, 95% CI: 6.09–81.54, *p* < 0.001) and those without insurance (OR = 3.95, 95% CI: 1.37–11.38, *p* = 0.011) reporting higher odds of affordability. Compared to government employees, lower odds of perceiving healthcare as affordable were observed in unemployed (OR: 0.605, 95% CI: 0.151–2.419, *p* = 0.015) and self-employed individuals (OR: 0.266, 95% CI: 0.055–1.286, *p* = 0.032). Lastly, individuals who utilized healthcare services in the past year had lower odds (OR = 0.34, 95% CI: 0.16–0.72, *p* = 0.006) of perceiving healthcare as affordable.

Chi-square tests revealed that the distance to healthcare facilities was significantly associated with living situation (χ² = 73.2, *df* = 6, *p* < 0.001) and duration of displacement (χ² = 54.6, *df* = 8, *p* < 0.001). Those in public buildings or host communities reported closer proximity to healthcare, while longer displacement durations were associated with greater perceived distance. Transportation availability also significantly influenced healthcare utilization (χ² = 23.7, *df* = 4, *p* < 0.001), with individuals relying on walking or public transport more likely to have sought healthcare in the past year.

The availability of qualified staff was a critical facilitator of healthcare utilization. A significant relationship was observed between the availability of qualified staff at healthcare facilities and the likelihood of seeking medical services (χ² = 11.30, *p* = 0.022). Those who reported “Always” having access to qualified staff were significantly more likely to have sought medical services in the past year compared to those who reported “Never” or “Sometimes”. 

Quality of life domains showed significant variation by both the duration of displacement and living situation. Individuals displaced for 3 to less than 6 months reported better physical well-being (mean = 51.2, *F*(4,568) = 14.18, *p* < 0.011), while those displaced for over 12 months had significantly poorer psychological well-being (mean = 45.8, *F*(4,585) = 10.91, *p* < 0.011). Social well-being was highest among those displaced for 9 to less than 12 months (mean = 50.0, *F*(4,502) = 4.26, *p* < 0.011). Regarding living situations, residents of host communities exhibited the highest physical well-being scores (mean = 53.6, *F*(3,577) = 14.34, *p* < 0.011) and psychological well-being scores (mean = 58.3, *F*(3,596) = 12.49, *p* < 0.011). Social well-being was notably better among residents in public buildings and host communities compared to displacement camps (*F*(3,502) = 4.86, *p* < 0.011). Environmental well-being did not show significant differences across either displacement durations (*F*(4,562) = 1.65, *p* = 0.161) or living situations (*F*(3,572) = 1.20, *p* = 0.311).

Healthcare utilization in the past year was significantly linked to psychological and environmental well-being. Individuals who sought medical services showed better environmental well-being (*F*(1,566) = 6.22, *p* = 0.013) and a trend toward higher psychological well-being (*F*(1,589) = 3.82, *p* = 0.053) compared to those who did not access healthcare. However, no significant differences were observed for physical (*F*(1,572) = 0.34, *p* = 0.563) or social well-being (*F*(1,505) = 1.41, *p* = 0.243).

## Discussion

During the Sudan conflict and subsequent mass displacement, many internally displaced people (IDP) faced significant barriers to accessing healthcare services. These barriers led to delays in treatment and, in many cases, preventable deaths.

### Healthcare accessibility and utilization

For the last couple of years, access to healthcare in Sudan was defective even before the war. Patients faced accessibility barriers, related but not limited to: services availability, high cost, transportation, and lack of qualified staff [20]. More specifically, long waiting time and distance were reported as a major barrier to receiving care in people with chronic conditions [21, 22], along with stigma and health providers attitude [23]. However, the perceived level of accessibility to essential medicine in Khartoum State at primary care level was more adequate, where average waiting time was 21.1 min and patients were treated with courtesy, yet distance to PHC centers was still a remaining issue [24].

Concerning healthcare access and utilization, we found that 11.5% of IDP actually perceived health facilities as too distant to reach. This finding is consistent but slightly less than what was reported by international organization for migration (IOM) in the last bi-monthly mobility overview, where 14% of IDP perceived healthcare available but too far away [25].

Regarding waiting times, only 13% of participants in our study reported waiting for more than 2 h to receive care, compared to 56% in Northern Uganda [19]. While this finding might initially suggest better efficiency in Sudanese healthcare services, it is more likely reflective of the lower utilization of these services due to accessibility issues and resource constraints. The reduced demand on healthcare facilities could inadvertently result in shorter waiting times, but it highlights the underlying issue of limited access and unmet healthcare needs among the displaced population.

Half of the IDP found healthcare to be unaffordable, with a similar proportion reporting a lack of health insurance coverage. Additionally, 62.5% reported delaying or avoiding healthcare due to financial constraints. This is similar to IDP in Bogotá, Colombia, where the cost of health services is the main issue, and most IDP fall in the lowest tier of health insurance, which severely impairs their health care access [26]. 45% of the IDP sometimes found qualified healthcare staff. This percentage is concerning, as the presence of staff contributes to improvements in health outcomes and ultimately extend the life expectancy of populations [27].

In our study, the largest percentage of IDP receive healthcare from public health facilities supported by non-governmental organizations (NGOs). This context is similar in African countries such as Uganda and the Democratic Republic of Congo, where collaborations between government health sectors and humanitarian agencies have ensured better healthcare access for displaced populations [28].

Most IDP obtained their prescribed medications from local pharmacies or healthcare facilities. Similarly, in other contexts, IDP frequently acquire their medications either through local pharmacies or donated drug supplies made accessible at healthcare facilities serving displaced populations [29, 30]. This highlights the critical role of local dispensaries and donated drugs in ensuring access to essential medications for vulnerable populations.

The availability of healthcare staff played a crucial role in facilitating healthcare utilization among IDP. A significant association was found between the presence of qualified staff at healthcare facilities and the likelihood of seeking medical services. This aligns with the findings of the WHO program on increasing access to health workers in remote and rural areas through improved retention, which highlighted how the lack of qualified health workers limits access to essential services [31]. This highlights that healthcare utilization might be shaped by the trust and confidence patients have in skilled staff. Similarly, a study among IDP in Nigeria identified poor staffing as a major barrier to accessing care [32], highlighting the widespread challenges caused by inadequate human resources training and distribution in healthcare during crises.

Females were found to face more financial barriers to healthcare services compared to males. This is consistent with observations in the general population, where research has shown that women often experience greater financial strains [33]. This disparity may stem from gender-based inequities in income, employment opportunities, and societal roles, which limit women’s ability to afford medical care. Such challenges can further perpetuate poor health outcomes among women, particularly in vulnerable settings.

The findings suggest that individuals who are unaware of their health insurance coverage, as well as those without insurance, are more likely to perceive healthcare as affordable. This could indicate that these groups may have a higher sense of financial security or less awareness of the actual costs of healthcare. People who lack insurance or aren’t informed about their coverage might not fully understand the potential financial burden of healthcare, leading them to believe it is more affordable than it actually is. This perception might also reflect a sense of economic comfort or detachment from the complexities of healthcare costs. Additionally, employment status emerged as a key determinant of affordability, with unemployed or self-employed individuals being less able to afford healthcare services. This highlights the economic vulnerabilities faced by these groups, where irregular or insufficient income creates significant barriers to accessing necessary care. It reflects how structural factors, such as job security and economic stability, directly influence the individual’s ability to prioritize healthcare.

### Quality of life

Regarding the quality of life among IDP, our study found lower psychological, social, and environmental quality of life scores compared to IDP in Iraq [34], though the physical domain was slightly higher. These differences may be influenced by the limited availability of humanitarian aid in Sudan [35] and the country’s unstable socio-political environment. Applying Andersen’s Behavioral Model, the higher physical quality of life scores in Sudan could be attributed to the resilience of IDP in meeting basic physical needs despite systemic challenges, whereas psychological and social domains may be more affected by prolonged displacement and lack of social support systems. Similarly, the Social Determinants of Health framework highlights how environmental and socio-political instability disproportionately impacts mental and social well-being, while physical health may be temporarily sustained through community coping mechanisms. Further investigation is essential to better understand these disparities and to address the specific challenges faced by IDPs in Sudan effectively.

Our findings reveal that displacement duration and living situations significantly affect quality of life (QOL) domains. Shorter displacement durations were linked to better physical well-being, reflecting resilience in early displacement, while prolonged displacement correlated with poorer psychological well-being, emphasizing the mental toll of long-term instability.

Living in host communities was associated with higher physical and psychological well-being, likely due to better resource access and social integration. However, environmental well-being showed no significant differences, suggesting uniformly poor conditions across all settings.

Healthcare utilization was positively associated with psychological and environmental well-being, emphasizing its role in enhancing mental health and perceptions of stability. However, the lack of impact on physical and social well-being underscores the need for broader interventions targeting these dimensions.

Moreover, there is a notable scarcity of literature exploring the association between quality of life among internally displaced people (IDP) and factors such as displacement duration, living conditions, and healthcare access. This gap in research limits our understanding of how these variables interact to influence QOL and highlights the need for future studies to address these relationships comprehensively. Filling this gap is crucial for designing effective, evidence-based interventions tailored to the diverse needs of displaced populations.

This study has also discussed the reasons behind why IDP may seek medical care and their choice of health facility over the other, in addition to the challenges in accessing healthcare and consequences of inaccessibility, and the alternative solutions they sought to meet their medical needs.

### Health seeking behaviours

Highlighting the main reasons leading IDP to seek medical care, we found that acute conditions such as infections and malaria were the biggest reason for IDP to seek medical attention by 68.5%. Other conditions such as chronic diseases were reported less. IDP priorities revolve around basic needs and security, such as livelihood and protection from violence and harassment, as well as access to essential food and sanitary water. According to a multi-sectoral needs assessment conducted by the international organization for migration (IOM), in the central region of Cameroon, which has an estimated 56,050 IDP; the priority needs of IDP emerge as: access to income-generating activities (60%), access to food (16%), and access to education for children (10%) [36]. In Sudan, the fight for accommodation and livelihood is even more challenging as the number of official IDP camps is fewer compared to hosting communities, rented accommodations, and public buildings as schools. According to Sudan’s Humanitarian update (1–30 November 2024) by OCHA, Over 97% of IDP across Sudan are in areas with high levels of acute food insecurity, and 89% of displaced families are unable to afford their daily food requirements [37]. Nevertheless, infectious disease and specifically malaria, diarrheal disease, and respiratory infections remain the main cause of mortality among IDP, due to camps overcrowding and unsanitary livelihood access [38]. It is also significant to mention that all these diseases are preventable, where water, sanitation, and hygiene, and well-conditioned livelihoods, are key factors in prevention.

Concerning the choice health facilities, the proximity of the facility to where the IDP reside emerged as a significant factor in determining which healthcare facility they might visit, as reported by 52.6% of them. Meanwhile availability of medications, specialized services, and free services constituted relatively similar percentages, at 24.8%, 21.8%, and 21.5%, respectively. These factors are important, as IDP experience hardships in health cost, as well as obtaining prescribed medication; hence the availability of all these factors is a determining factor [39]. Additionally, in a study that investigated the health-seeking patterns among IDP in Northeast Nigeria, 75.7% of them sought facility care over non-facility/home care. One of the significant reasons why IDP favored home care over facility care is residing over two kilometers away from the nearest health facility. Other factors influencing health seeking behaviors towards facility care included perception of illness severity, status of IDP camps, duration of residence in IDP camps, and access to disease surveillance information were all significantly associated with health-seeking patterns [40]. Moreover, IDP in northern Uganda reported that the main reasons for choice of health facility are proximity of health facility (29.6%), availability of free treatment(22.7%) [19], which is in favor of our results.

### Barriers in accessing healthcare services

Regarding barriers to accessing healthcare services, the challenges were themed into either challenges before reaching the facility and those after arriving at the facility like cultural barriers and applied stigma by the healthcare providers. Lack of transportation to healthcare facilities emerged as the most significant challenge faced by the IDP (37.9%). We also conducted a chi square test to investigate the association between healthcare utilization and availability of transportation, which significantly influenced health-seeking behaviors of IDP. Similarly, a qualitative study conducted in Afghanistan by Yagana Parwak et al. revealed that distance and transportation cost were significant barrier, especially after the discontinuation of transportation voucher services provided by local NGOs [39]. According to a global survey conducted by the Norwegian Refugee Council in November 2021 across 25 countries including Syria, Sudan, Ukraine, and Palestine, the main obstacles to accessing healthcare included distant health service, reported by 71% countries, in addition to transportation and movement restrictions, which were reported in Sudan and other countries [41]. Those results showed that IDP face obstacles in accessing healthcare, even before arriving at the healthcare facility, where distance, transportation, and its cost constituteahuge challenges. Our results highlighted barriers such as discrimination and stigmatization by healthcare providers (7%) and cultural barriers (5.7%). Though reported by a smaller proportion, these barriers have significant implications. Cultural stigmatization, tribal affiliations, and belief systems can deter healthcare-seeking behavior, particularly in context where trust in formal systems is low. Addressing these issues requires culturally sensitive interventions, such as training healthcare providers to mitigate biases and engaging community leaders to build trust.

Concerning alternative solutions sought by IDP to health inaccessibility, traditional remedies and at-home medicine were the most common options, reported by 60.3% of respondents. This finding is reasonable, especially given that traditional medicine holds cultural and historical value in the Sudanese community [42], as well as it is one of the coping mechanisms against healthcare inaccessibility in IDP context, alongside travelling to nearby areas [43]. Understanding whether this reliance on traditional medicine is driven by preference or sole necessity is critical for designing interventions, such as partnerships between traditional healers and formal providers, to ensure safe, complementary care.

With all this being mentioned, we need to highlight that 27.4% of our population indicated they face no challenges in accessing healthcare services, which might underscore disparities in effectiveness of the effort being made under this burdened system, among various states.

Weencountered problems in the data collection measures due to the sensitivity of the target group. Owing to the country’s economic circumstances, we were unable to secure funding for the research, which limited our ability to access a larger population or implement a systematic random sample. As a result, we had to rely on convenience sampling, leading to an unbalanced distribution of data throughout the targeted states. Additionally, the absence of official IDP records in camps and communities further hindered our ability to obtain accurate and comprehensive data, necessitating the use of convenience sampling as an alternative. Furthermore, Data collection was prohibited insome IDP camps and communities, due to security concerns, which compromised the safety of data collectors and leading to reduction in sample size, making it not fully representative of the entire IDP population in Sudan.

We recommend conducting research to evaluate the effectiveness of economic empowerment programs for IDP and the utilization of voucher systems to address transportation and accessibility challenges, particularly for those displaced from remote areas. Future studies should also focus on assessing and monitoring the functionality of international non-governmental organizations (INGOs) operating in healthcare delivery through third-party evaluations. Additionally, longitudinal research is needed to monitor the long-term health outcomes of IDP and evaluate the impact of various interventions on their well-being. Studies should explore the specific needs of vulnerable groups within the IDP population, such as women, children, and individuals with disabilities, while investigating innovative approaches to improving healthcare access and utilization. This approach could include telemedicine, mobile health services, and traditional medicine as cost-effective solutions. Expanding health insurance coverage to better include IDP populations should also be explored as a potential strategy for enhancing healthcare access.

## Conclusion

This study investigated healthcare access and utilization among internally displaced people (IDP) in Sudan, examining factors influencing their healthcare-seeking behavior and the impact of unmet healthcare needs on their quality of life. Key findings revealed significant challenges in accessing healthcare, including affordability, transportation barriers, and limited availability of qualified healthcare personnel. Displacement duration and living conditions further exacerbated these issues, with longer-term displacement and camp residency linked to poorer access, higher healthcare costs, and lower quality of life.

To address these challenges, actionable strategies should focus on context-specific interventions. Deploying mobile clinics and community health workers can help deliver essential services such as vaccinations, maternal care, and treatment for infectious diseases in remote and underserved areas. Implementing voucher systems for transportation and healthcare services can reduce out-of-pocket expenses, while cash transfer programs can alleviate financial barriers for vulnerable groups. Strengthening local health systems through facility rehabilitation, training of healthcare workers, and integrating mental health services into primary care are critical to improving the quality and sustainability of care. Additionally, expanding health insurance coverage and leveraging partnerships with NGOs and international organizations can enhance access to essential medications and specialized care.

Future research should evaluate the effectiveness of these interventions, particularly their adaptability to different displacement contexts. Studies should also focus on the specific needs of vulnerable groups, such as women, children, and individuals with disabilities, and assess the long-term impact of these strategies on health outcomes. By adopting a targeted and evidence-based approach, policymakers and healthcare providers can design more effective solutions to improve healthcare access and utilization for IDP in Sudan.

## Electronic supplementary material

Below is the link to the electronic supplementary material.

Supplementary Material 1

## Data Availability

The datasets used and/or analyzed during the current study are available from the corresponding author on reasonable request.

## Abbreviations

IDP: Internally Displaced People
WHOQOL-Bref: Abbreviated World Health Organization Quality of Life Questionnaire
SAF: Sudanese Armed Forces
RSF: Rapid Support Forces
QOL: Quality of Life
WHO: World Health Organization
NGOs: Non-Governmental Organizations
INGOs: International Non-Governmental Organizations
IOM: International Organization for Migration
OCHA: Office for the Coordination of Humanitarian Affairs
