# Supplementary material for: Healthcare accessibility, utilization, and quality of life among internally displaced people during the Sudan war: a cross-sectional study
Source: Confl Health. 2025 Mar 1;19:11. doi: 10.1186/s13031-025-00655-3 (PMC11871592; doi:10.1186/s13031-025-00655-3)
Supplement: Supplementary file 1 — Supplementary Material 1 [file 13031_2025_655_MOESM1_ESM.docx]

**Supplementary file 1: Demographic characteristics of participants (continued)**

|  | **Overall (N=612)** |  | **Overall (N=612)** |
| --- | --- | --- | --- |
| **Highest Educational Level Attained** |  | **Household Size** |  |
| Illiterate | 29 (4.8%) | Mean (SD) | 6.9 (2.7) |
| Intermediate School | 40 (6.7%) | Range | 0.0 - 17.0 |
| Primary School | 32 (5.3%) | **Pregnant Women or Children Under Five in the Household** |  |
| High School | 220 (36.7%) | No | 320 (53.0%) |
| Informal Education (Khalwa) | 28 (4.7%) | Children Under Five | 168 (27.8%) |
| Vocational/Technical Education | 43 (7.2%) | Pregnant Women | 30 (5.0%) |
| Bachelor's Degree | 175 (29.2%) | Both | 86 (14.2%) |
| Doctorate | 3 (0.5%) | **Current Employment Status** |  |
| Master's Degree | 13 (2.2%) | Government Institution | 63 (10.3%) |
| Fellowship | 16 (2.7%) | Private Institution | 26 (4.3%) |
|  |  | Unemployed | 373 (61.1%) |
|  |  | Self-Employed | 119 (19.5%) |
|  |  | Business Owner | 29 (4.8%) |
